# (How) is COVID-19 reframing interaction between the NHS and private healthcare?

**DOI:** 10.1177/09685332231159362

**Published:** 2023-03-14

**Authors:** Mary Guy

**Affiliations:** Liverpool John Moores University, UK

**Keywords:** Competition, COVID-19, dual practice, National Health Service (NHS), NHS England, Independent Healthcare Provider Network, private healthcare, Competition Act 1998 (Health Services for Patients in England) (Coronavirus) (Public Policy Exclusion) Order 2020, Competition Act 1998 (Health Services for Patients in Wales) (Coronavirus) (Public Policy Exclusion) Order 2020, The Competition Act 1998 (Health Services for Patients in England) (Coronavirus) (Public Policy Exclusion) Order 2022

## Abstract

In March 2020 a ‘major deal’ was struck between the National Health Service (NHS) and private healthcare sector to facilitate ‘crisis’ and ‘continuity’ responses to COVID-19. A further deal was struck in January 2022 to support the NHS in tackling the Omicron variant, suggesting that the pandemic was evolving, rather than definitively over. The legal basis for these deals was a Public Policy Exclusion Order, a temporary relaxation mechanism in UK competition law defined by a ‘disruption period’. In a global pandemic, the ‘healthcare disruption period’ might be considered to be of a different scope and nature to short-term disturbances experienced in other sectors, such as groceries. This article examines the Public Policy Exclusion Orders issued in respect of health services in England and Wales, and the Collective Agreements notified under these between March 2020 and March 2021, and again in March 2022. Amid ongoing tensions surrounding ‘NHS privatisation’, this enables a timely analysis of whether the underlying relationship between the NHS and private healthcare may be changing in response to COVID-19, and how considerations of ethical frameworks are also relevant to this aspect of the pandemic response.

## Introduction

The interaction between the National Health Service (NHS) and the smaller, supplementary private healthcare sector is a contentious aspect of UK healthcare, affecting treatments as diverse as dentistry^1^ and in vitro fertilisation (IVF).^2^ The contentious aspect is evident in the private healthcare sector being considered: ‘ . . . to be responsible for setting trends for the allocation of healthcare resources and for upholding certain elements of justice . . .’;^3^ to contribute to perceptions of ‘NHS privatisation’,^4^ and even to impact the doctor–patient relationship.^5^ The interaction has existed since the inception of the NHS in 1948, and at its best is collaborative, but it also underpinned successive competition reforms in England. These culminated in the Health and Social Care Act 2012 (HSCA 2012)^6^ prior to the current focus on integrated care systems, now enshrined in law by the Health and Care Act 2022 (HCA 2022).

Responding to COVID-19 highlighted the potential for collaboration between the two, at the levels of what might be termed ‘crisis response’ and ‘continuity’ (with regard to re-starting non-COVID-19 healthcare services). In March 2020, a ‘major deal’ was struck between the NHS and the Independent Healthcare Provider Network (IHPN).^7^ This involved transfer of all private healthcare facilities to support the NHS in the initial pandemic response, as well as evolving interactions to tackle backlogs and provide ongoing support subsequently. In England, this was particularly wide-ranging, given the varying NHS structures and expansion of private healthcare relative to the other countries of the United Kingdom,^8^ but formal arrangements were also put in place between the Welsh Health Specialised Services Committee and the IHPN, despite the different level of interaction between the NHS and private healthcare sector in Wales.^9^

A further deal was signed in January 2022 ‘under direction from the Secretary of State’ between NHS England and 10 private healthcare providers to enable local hospitals to activate surge capacity quickly as part of a national COVID-19 response.^10^ This appears to relate to the government’s triggering of ‘Plan B’^11^ to support the NHS with regard to the Omicron variant of COVID-19, which emerged in late 2021.

What gave effect to these ‘deals’ was a relaxation of competition law permitting agreements between NHS and private providers that might otherwise be deemed anticompetitive. This legal basis is found in the Competition Act 1998 (Health Services for Patients in England; Coronavirus; Public Policy Exclusion) Order 2020^12^ (the 2020 Order), with an equivalent order containing many identical framings being issued in respect of patients in Wales.^13^ Both orders were revoked in July 2021,^14^ clearly preceding the acknowledged need to re-expand independent sector support to the NHS in tackling the Omicron variant in December 2021.^15^ The 2022 Order was introduced in England briefly,^16^ apparently to tackle the backlog that was exacerbated (rather than caused) by the pandemic, yet this issue too is ongoing beyond the 2022 Order timeframe.^17^ In contrast to the separate 2020 Orders for England and Wales, the 2022 Order references ‘the UK’ (yet appeared primarily focused on England).

By examining the 2020 and 2022 Orders and their implementing collective agreements, it becomes possible to gain insights beyond pre-pandemic knowledge of the contours of the relationship between NHS and private providers. This relates, on one hand, to New Labour policy and HSCA 2012 reforms enabling expansion of private providers delivering NHS services and, on the other hand, to ‘dual practice’, whereby healthcare professionals treat both NHS and private patients. While this latter phenomenon is a long-standing feature of the NHS,^18^ it is surprisingly under-researched, particularly within the health law context.^19^ Nevertheless, it has clear implications beyond the context of UK healthcare, with various other countries integrating public and private healthcare, and engaging with the challenges posed by claims of ‘two-tier’ healthcare access.^20^

As questions are raised about the benefits of this ‘major deal’,^21^ this article provides a timely examination of the 2020 and 2022 Orders as mechanisms put in place to respond primarily to the COVID-19 pandemic by facilitating NHS–private healthcare interaction. It juxtaposes these with the pre-pandemic relationship to evaluate whether the interaction may be changing given the myriad challenges faced by the NHS in early 2023. ‘The section “four categories of English healthcare” - before and during the COVID-19 pandemic’ outlines NHS–private healthcare interaction by reference to ‘four categories’^22^ to provide the necessary context to this discussion. This is then developed via two lenses drawing on pandemic-related narratives. The section ‘“Business as usual”? Path dependency and NHS–private healthcare interaction’ looks behind claims of ‘business as usual’ to examine whether path dependency can explain whether the COVID-19 pandemic can shape current and future NHS–private healthcare interaction. The section ‘“Learning to live with COVID-19”: a shifting ethical framework for NHS–private healthcare interaction?’ then engages with considerations emerging about changes to the ethical framework underpinning healthcare access in ‘learning to live with COVID-19’. The final section offers some ‘Concluding Remarks’.

## The ‘four categories of English healthcare’ – before and during the COVID-19 pandemic

**Figure 1.**
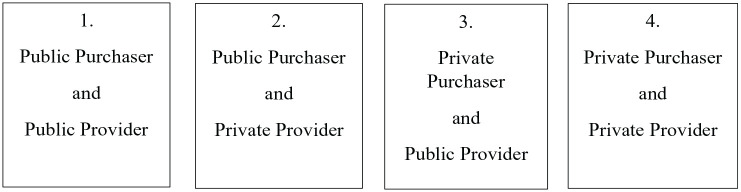
Relationship between the NHS and private healthcare sectors as demonstrated by the purchaser/provider separation.

One way to understand the interaction between the NHS and private healthcare is as ‘four categories’,^23^ comprising the distinction between the NHS (categories 1 and 2) and the private healthcare sector (categories 3 and 4) and the separation of purchasing and providing functions that have underpinned the competition and marketisation reforms in the English NHS. This can be illustrated as shown in Figure 1.

In general terms, category 1 refers to NHS patients being treated by NHS providers (e.g. NHS Trusts/Foundation Trusts); and category 2 encompasses NHS patients being treated by private providers. The private healthcare market is made up of category 3, representing private patients being treated by NHS providers (in private patient units) and category 4, consisting of private patients being treated by private providers.

The four categories can be summarised by two framings. First, ‘going private’, which reflects a change in status from NHS patient to private patient (thus moving from categories 1 and 2 to categories 3 and 4). Second, and in contrast, ‘NHS privatisation’, which generally^24^ refers broadly to the expansion of private sector delivery of NHS services to NHS patients (category 2), developed by New Labour and, subsequently, as a response to lengthy waiting lists and increasing patient demand.

The aforementioned contentious aspect in the long-standing NHS–private healthcare interaction is created by the possibility for healthcare providers to work in both the NHS and private healthcare sector. The contentiousness can be illustrated by reference to the prohibition on ‘co-funding’, which is intended to avoid even the perception of the NHS subsidising private healthcare, in policy guidance on where patients can access both NHS and private treatment, provided these are kept separate.^25^ This need for separation has evolved over time to facilitate payment for cancer drugs (not yet approved for the NHS) by patients receiving cancer treatment on the NHS.^26^ However, there are also treatment areas that become characterised by ‘private absorption’ of (NHS) patients in response to limited availability of NHS services, such as dentistry,^27^ a sector which has seen successive reforms with personal responsibility components thought capable of precluding access to NHS dentistry and exacerbating health inequalities.^28^ Another example would be differing approaches taken to rationing IVF treatment at a local level in England,^29^ but more generally across the United Kingdom, with the Human Fertilisation and Embryology Authority indicating declines in NHS funding in England and Northern Ireland in particular.^30^

Over time, expansion of private sector activity with regard to specific treatments has prompted further perspectives to emerge as consumer law and competition law become more relevant, prompting formal interest to be shown by the competition authority,^31^ alongside oversight by other regulators, notably the Care Quality Commission (CQC). The private healthcare sector market has been defined by the Competition and Markets Authority (CMA) by reference to the NHS, with NHS work forming part of the business plan of some private healthcare providers (which thus may feature in both categories 2 and 4), but not others^32^ (which exclusively operate in category 4). This dynamic has appeared more pronounced in response to external factors, such as the welcoming of NHS work by private providers during the economic downturn of 2008–2009.^33^ Regarding the reach of competition law, and specifically the prohibition on anticompetitive agreements,^34^ controversy has arisen in respect primarily of the (theoretical) applicability of competition law in respect of category 2 activity, although enforcement action by the competition authority has focused on the private healthcare market (categories 3 and 4).^35^ The HSCA 2012 competition reforms, however, took a largely different focus, comprising a ‘NHS-specific’ competition regime largely overseen by NHS Improvement, as distinct from the national general competition law framework of the Competition Act 1998.^36^

It is against the backdrop of these dynamics that responses the COVID-19 pandemic emerged, including further welcoming of NHS work by private providers during the initial lockdown.^37^ To facilitate cooperation between private and NHS providers in responding to COVID-19, there was a need to remove certain agreements formally from the scope of competition law, as these may ordinarily be considered in breach of the Competition Act 1998.

The mechanism used to achieve this result was a Public Policy Exclusion Order (PPEO), which is intended to provide a limited, and where possible, time-bounded, relaxation of the competition rules by reference to a ‘disruption period’ defined by the Secretary of State for Business, Energy and Industrial Strategy in specified circumstances, if satisfied that there are exceptional and compelling reasons of public policy.^38^ In the initial COVID-19 response phase, PPEOs were also implemented in respect of groceries,^39^ dairy products,^40^ and Solent crossings.^41^ These were broadly in operation between March/April 2020 and September/October 2020, when the relevant ‘disruption period’ was deemed to be at an end.

‘PPEOs may make sense in the contexts particularly of groceries and dairy products where supply chain interruptions may occur periodically, for example, as a result of ‘panic buying’. However, the logic of this particular mechanism in the healthcare sector to respond to, and move beyond, a global pandemic is arguably less persuasive. This is because the shock experienced by the healthcare sector as a result of the COVID-19 pandemic was of a different scope and scale to that experienced by other sectors. Indeed, in view of the evolving nature of the pandemic during 2020 and 2021 in particular, which encompassed both the emergence of new variants of COVID-19 and governmental attempts to curb the spread of the virus via successive lockdowns and mass vaccination programmes, it may be difficult to see how a ‘*healthcare* disruption period’^42^ could be anything other than open-ended. Nevertheless, the PPEOs relating to English and Welsh healthcare were in operation broadly from March 2020 until they were revoked in July 2021,^43^ while an additional PPEO for English healthcare was introduced for a period of mere weeks in March 2022.

This framing of ‘healthcare disruption period’ is clearly dictated by the nature of the legal instrument, but offers a distinctive perspective on the different phases of the pandemic. A growing literature identifies different taxonomies, comprising, for example, ‘response’ and ‘recovery’ phases,^44^ ‘contingency’,^45^ ‘reset’,^46^ and rightly highlights the difficulty of framing phases in a meaningful way.^47^ In contrast, a ‘healthcare disruption period’ may appear at once simplistic and difficult to define, as well as suggesting a formalistic approach, which cannot engage with the complexity of issues that may comprise ‘healthcare disruption’.

As noted above, the ‘major deal’ struck between the NHS and private healthcare was announced in March 2020. Accordingly, the 2020 PPEOs for both England and Wales back-date the start of the ‘healthcare disruption period’ to 1 March 2020,^48^ and stipulate that this was not anticipated to be shorter than 28 days.^49^ The expiration of contractual agreements was also confirmed retrospectively as 31 March 2021.^50^ This might appear to suggest that closer cooperation between the NHS and private healthcare was in operation for approximately 1 year, with the potential to distort the ‘four categories’ framework. However, while, during the initial lockdown, the focus was undoubtedly on supporting the NHS in treating COVID-19 patients, residual scope for urgent non-COVID-19 treatment of private patients emerged.

The 2020 Orders for England (and Wales) permitted five kinds of agreements: sharing information about capacity to provide certain services; coordination on deployment of staff; sharing or loan of facilities; joint purchasing of goods, facilities, or services; and division of activities, including agreement to limit or expand the scale or range of services supplied by one or more providers.^51^ The need for cooperation between the NHS and independent providers to provide extra capacity and meet local needs in responding to COVID-19 was underscored.^52^ These agreements could be between independent providers (arguably suggesting a focus on either category 2 or category 4 activity), and between independent providers and NHS bodies (which may suggest a focus primarily on category 2 activity). The stipulation that agreements must not involve the sharing ‘between independent providers of any information regarding costs or pricing’^53^ might seem to lend support to the view that category 4 activity was targeted given the limited scope for price competition in category 2 as a result of the National Tariff, which determines price of NHS treatments.

While the 2020 Orders for England and Wales were ultimately time-bounded (March 2020 to July 2021), the Collective Agreements (which implemented them) reflect a more phase-based approach. Thus initial agreements were signed in April 2020, with later agreements (for England only) signed in September and December 2020, then again in March 2022 (to support the 2022 Order). For the purposes of the current discussion, these agreements can be grouped under the broad headings of ‘crisis’ and ‘continuity’ responses.

### The ‘Crisis’ Collective Agreements (England and Wales)

The Collective Agreements for the crisis period for England^54^ and Wales^55^ comprised a focus on secondary care services, such as ‘provision of full hospital capacity and services including acute bed capacity . . . facilities, diagnostics, staffing, management and full organisation capability’. In contrast, primary care services and community services were not included within the agreement, even if co-located with hospital services.

A notable aspect of the agreements was that the private providers would comply with NHS rules for cancellation and prioritisation of elective care from a specified date.^56^ After this date, private providers appeared still limited in the range of private patients who could be treated (e.g. urgent oncology cases and long-term neurological conditions). The agreements further envisaged that the service offered by private providers would encompass three main aspects. First, inpatient and outpatient urgent elective and cancer treatment in line with nationally set criteria to offset reduced capacity so that NHS could focus on the most acute cases. Second, inpatient non-elective care and converting day-case-only facilities to support this. Finally, providing NHS care for COVID-19-infected patients needing high dependency respiratory support on oxygen therapy and non-invasive ventilation (NIV) therapy.^57^

In elaborating the implementation of these agreements, we started to see differences in the framing of the English and Welsh approaches. For example, ‘operational flexibility’ was required in Wales ‘where possible’, but was linked in England with a ‘peak surge’ period, suggesting a focus on providing ‘equipment and stock in providing care’. A further distinction can be seen in the scope of the Operational Agreements under the English ‘crisis’ agreement acknowledging a role for private medical insurers (PMIs) to comment on the impact on the private healthcare market.

Overall, it is difficult to obtain a clear picture of the range of providers involved in the English and Welsh agreements. Certainly, the 27 ‘participating IHPN members’ in the English ‘crisis’ agreement may seem a small number relative to the 65 listed as members of the IHPN.^58^ However, those listed as participating in the English agreement appear to include wider provider groups (such as Spire Healthcare Limited or BMI Healthcare Limited) as well as seemingly smaller, individual providers (such as The London Clinic). The range of NHS Trusts and NHS Foundation Trusts listed as participating in the agreement appears broadly consistent with wider lists.^59^ Similar queries might be considered to emerge in connection with the range of IHPN members participating in the Welsh ‘crisis’ agreement – with five wide groups (such as Spire Healthcare Limited and Nuffield Health) listed in contrast to the 12 listed as IHPN members.^60^ All three NHS Trusts and the seven Local Health Boards in Wales^61^ were party to the agreement.

### The ‘Continuity’ Collective Agreements for supporting provision of elective care in England

Two broad categories of ‘continuity’ agreements can be identified, emphasising, respectively, continuity of private and NHS elective care in England.

First, an agreement was concluded with the purpose to ‘ . . . collectively agree and implement a process for the use of surplus capacity within the independent sector provider facilities to specifically be used for private medical insurer (PMI) funded and privately-funded urgent elective care’.^62^ The parties to this agreement were the 27 IHPN members who also joined the original English ‘crisis’ agreement. This perhaps indicated an intention to continue private work (in categories 3 and 4) alongside NHS work from a relatively early point in the pandemic response, supported by an early triggering of the ‘de-escalation clause’ in May 2020, during the initial block-booking phase, to enable negotiations of how to support escalating waiting lists.^63^

Following calls for ongoing support from private healthcare in delivering non-COVID-19 services during summer 2020,^64^ continuity of NHS elective care was also addressed by Collective Agreements to underscore private healthcare sector support of the NHS in September 2020^65^ and December 2020,^66^ with the latter indicating an inclination to return to ‘business as normal’ from 1 April 2021. With the rapid spread of the ‘Kent’ variant of COVID-19,^67^ imposition of the third national lockdown and the escalation of deaths, which emerged throughout December 2020 and January 2021, concerns about the scope and interpretation at local level of the latter agreement emerged over 2021.^68^

Distinctions emerge between the NHS ‘continuity’ agreements, with the first (September 2020) operating to serve a transitional ‘de-escalation’ phase beyond the initial ‘crisis’ response,^69^ the second (December 2020) offering ‘buffer capacity’ to help the NHS cope with healthcare pressures caused by the COVID-19 pandemic,^70^ and the third (March 2022) focusing on ‘directly or indirectly supporting the provision of services by NHS bodies to address coronavirus and coronavirus disease’.^71^ These distinctions have had implications for the range of private providers involved, although a ‘core’ may be identified who have been involved across the ‘crisis’ and ‘continuity’ agreements. The later two ‘continuity’ agreements can be linked with NHS England’s power to trigger surge activity, suggesting an important new dynamic to the NHS–private healthcare interaction, which is persisting beyond the initial phases of the pandemic.

Despite these myriad convoluted arrangements, it has been considered that, ‘in practice, very few COVID-19 patients were treated in private hospitals’,^72^ raising questions about whether the ‘deal’ was a good use of public money. The explanation for this finding is thought to be attributed in part to the existence of ‘dual practice’, meaning that the deal ‘often simply secured access to hospital buildings and equipment but without the staff to run them’.^73^ It has been further considered that the agreement meant that many private sector hospitals stood empty while private sector waiting lists were growing, and that doctors working exclusively in the private sector had their ability to work temporarily restricted.^74^

A curious distinction from the 2020 Orders is that while the focus of the 2022 Order is England, its extent covers all four countries of the United Kingdom (the 2020 Orders had specified, respectively, England or Wales). However, this might be explained by the finding that, due to closer cooperation during the pandemic, the relationship between the NHS and private healthcare in Wales is developing.^75^

Having outlined the focus and operation of these Collective Agreements under the 2020 and 2022 Orders, it is now possible to consider how these advance our understanding of evolving ethical frameworks and the persistence of NHS–private healthcare interaction.

## ‘Business as usual’? Path dependency and NHS–private healthcare interaction

Theories of ‘path dependency’ have been used to explain the entrenchment of NHS marketisation policies,^76^ but are also relevant to wider NHS–private healthcare interaction, both pre- and post-COVID-19. ‘Path dependency’ in this context might best be understood in terms of ‘theorizing how policy can become so institutionalised and historically embedded that it becomes nearly impossible to break free . . . ’,^77^ and of actors being so ‘hemmed in by existing institutions and structures that channel them along established policy paths’.^78^

These definitions go a long way to explaining how the interaction between the NHS and private healthcare has seen limited evolution since the inception of the NHS in 1948, in view of relatively stable governments, albeit with political shifts between Labour and Conservatives. This limited evolution of NHS–private healthcare interaction came about as a result of concessions of allowing medical professionals (notably consultants) scope to continue private practice alongside their NHS workload,^79^ supported by hospital accommodation being made available to treat private patients.^80^ This underpins the distinction between the possible shift in status between ‘NHS patients’ and ‘private patients’, and the subsequent political controversies such as removing ‘NHS pay-beds’ (by the Labour party in the 1970s).^81^ Indeed this contentious interaction between the NHS and private healthcare also provided a basis for ‘selling’ New Labour ‘choice and competition’ reforms of the early 2000s insofar as former Prime Minister Tony Blair described the expansion of private sector delivery of NHS services thus:

The overriding principle is clear. We should give poorer patients . . . the same range of choice [i.e. of a private provider] the rich have always enjoyed.^82^

This would appear to indicate not only that the marketisation reforms can be seen as a microcosm of the wider NHS–private healthcare interaction, but also that they represent a broad consolidation of a particular model. This would seem to support the view that it is ‘less remarkable that we often witness periods of continuity, and more so that change happens at all’.^83^ Despite wider distinctions between Labour and the Conservatives, notably in the 1980s and between 2015 and 2019, the interaction appears to remain largely intact, supported by the latest policy guidance on patient movement between the NHS and private healthcare dating from 2009.

It has been considered that ‘[t]he central problem of the path-dependency approach comes in explaining how policy change occurs, given the degree of entropy it hypothesizes’.^84^ Certainly, the persistence of NHS–private healthcare interaction prevails overall: notable changes arise in terms of the degree to which patients access private, rather than NHS healthcare. This appears linked to economic factors: hence, less uptake of private healthcare (and private medical insurance) at times when NHS spending is increased (e.g. under New Labour), or in response to clearly extrinsic factors, such as the economic downturn of 2008–2009.^85^

Nevertheless, significant changes do occur, but the timing appears key. Juxtapositions such as ‘structure and conjuncture’,^86^ with the latter illustrated as ‘windows of exceptional opportunity . . . that determine the ways small or big that a political system responds to policy imperatives’^87^ have been used to explain the introduction of the NHS internal market in the late 1980s. This move was made possible by the relative strength of the Thatcher government following the 1987 election and perceptions of responding to crisis in the NHS at that time.^88^

The NHS internal market was characterised by the separation of purchasing and providing functions to generate some degree of competition. While the ‘internal market’ terminology was abandoned by New Labour, this characteristic feature was retained, enabling not only the ‘choice and competition’ policy reforms, but also underpinned the subsequent legislative reforms regarding competition in the HSCA 2012. A decisive move away only occurred with the policy shift towards integrated care models from approximately 2015,^89^ and these are now enshrined in legislation by the HCA 2022.

Part of the controversy surrounding the HSCA 2012 reforms lies in the use of legislation rather than policy^90^ – which might be considered to entrench particular arrangements still further. Thus far, while the parameters of the NHS–private healthcare interaction framework have been set out in legislation, details of its operation have existed at the level of policy. There have been repeated attempts to redefine this operation in legislation with the ‘National Health Service (Co-funding and Co-payment) Bill’ introduced in almost every parliamentary session since 2017 by the Conservative MP Christopher Chope.^91^ This seeks to remove the current prohibition on co-funding and co-payment, facilitating access to, and delivery of, private healthcare alongside NHS services. If this proposal gains traction^92^ – an increasing possibility in the current climate of wide-ranging problems facing the NHS – it could herald a change more profound than the NHS internal market. However, whether all the necessary elements for ‘conjuncture’ are in place is moot.

### COVID-19 and path dependency

Against this backdrop of the very entrenched interaction between the NHS and private healthcare, the question is whether a global pandemic could be enough to trigger change. Here too, responses differ along the lines of ‘crisis’ and ‘continuity’ responses. Related to the aforementioned ‘structure and conjuncture’ framings, but ultimately distinct, is Kingdon’s ‘multiple streams’ framework and the ‘window of opportunity’ model.^93^ This requires the identification – and crucially, the coupling – of problem, politics, and policy streams to enable change (via a ‘window of opportunity’ and how wide, and for how long, this opens). This also offers an additional perspective on the extent to which the COVID-19 pandemic may effect significant change in the interaction between the NHS and private healthcare.^94^

Within such a framing, the problem stream comprises, naturally, the COVID-19 crisis response, the politics stream encompasses the Conservative government supported by the Labour and other opposition parties, and the policy stream represents the facilitation of greater NHS and private sector interaction. These streams can be considered to have coupled to the extent that the short-term response afforded by the ‘major deal’ was enabled. However, whether these streams can be said to converge in the same way for ‘continuity’ responses is moot. While there may (as at January 2023) be greater alignment between Labour and the Conservatives regarding use of the private healthcare sector in delivering NHS services, the nature of the problem stream is arguably long-standing and of longer duration, which makes coupling more difficult.

How the initial and subsequent responses to the COVID-19 pandemic relate to path dependency explanations is also difficult to pinpoint. On one hand, it might be considered that the initial crisis response – the ‘historic deal’ underpinned by the PPEOs and associated collective agreements covering both support for the NHS and temporary cessation of private healthcare delivery – indeed marked a change from the ‘path’ of separate NHS and private healthcare sectors, albeit with movement between the two. However, the inevitably temporary nature of a ‘crisis response’ would seem to suggest that any change would be one of degree rather than a fundamental shift. Thus, we see acknowledgement – even appreciation – of more NHS work among private practitioners during the initial lockdown.^95^ As with the responses to the 2008/2009 economic downturn, this may be considered short-lived. The choice of legal instrument – a PPEO – may be considered to lend support to the view that even a global pandemic may not be enough to displace the significant path dependence of the interaction between the NHS and private healthcare. Thus, notwithstanding initial shocks of the pandemic, the temporary nature of the PPEO alone may suffice to mean that the fundamental and familiar relationship would reassert itself sooner or later. This appears borne out thus far by the experience of the 2022 Order as a response to emergence and management of the Omicron variant of COVID-19 in late 2021.

In other words, the flexibility of this instrument may be testament to an underlying wish not to seek to alter or disrupt the fundamental interaction between the NHS and private healthcare, whereby patients may seek to move between the two. This flexibility might seem to offer a more targeted response whereby certain private providers increase, even prioritise, their NHS work during a specified, temporary period. Thus, the ‘continuity’ agreements may indicate a new dimension to path dependency – in other words, as new COVID-19 variants emerge, or simply in instances where the NHS risks being overwhelmed (e.g. by the ‘twindemic’ of influenza and COVID-19), there is now a precedent for recourse, which can be followed to ease excesses.

It remains particularly interesting and notable that a single set of agreements was signed between the NHS in Wales and the IHPN. This may, nevertheless, still be regarded as testament to the path dependence of NHS–private healthcare interaction, although a change may be afoot with closer NHS–private healthcare cooperation following the crisis response.

If the (general, rather than country-specific) path dependency of the NHS–private healthcare interaction is ultimately so entrenched that only a temporary divergence is possible, this prompts the question of what – if anything – could effect change. With this in mind, it might be considered that the COVID-19 pandemic may yet contribute to, but not be the sole cause of, a potentially seismic change in the interaction between the NHS and private healthcare.

## ‘Learning to live with COVID-19’: a shifting ethical framework for NHS–private healthcare interaction?

The outbreak of COVID-19 naturally also raised questions about the ethical frameworks underpinning treatment decisions, which also might be classed as falling broadly within ‘crisis’ and ‘continuity’ responses to the pandemic, albeit with scope for overlap between the two. These can be set against wider ethical concerns and frameworks relating to NHS and private healthcare interaction prior and subsequent to the pandemic.

While NHS–private healthcare interaction is but one aspect of wider discussions about healthcare delivery and reform, it is curious how little (explicit) attention it appears to receive in ethical considerations to date. This might be partially explained by what has been termed the ‘clinical’ and the ‘organisational’ levels of healthcare delivery. Between these two levels, a distinction appears evident between those for whom it is quite apparent ‘ . . . that ethical questions on the clinical level lead directly to organizational questions and that the two cannot be separated’,^96^ and others for whom ‘ . . . it may appear that there is no need for a separation of interests here because the same ethical principles apply to both levels’.^97^ However, regardless of the extent of this explanation, there is a need to locate questions about ethics, which relate to NHS and private healthcare interaction in general, and then, more specifically, with regard to COVID-19 responses.

### General considerations: ethical concerns and NHS–private healthcare interaction

Concerns about expansion of private healthcare are multi-faceted, with a particular fear being that ‘ . . . [privatisation and market forces] . . . will inevitably undermine the ethical foundations of medical practice and dissolve the moral precepts that have historically defined the medical profession’.^98^ Interest in the limits of markets in healthcare is not, however, confined to bioethicists and health lawyers. The idea that a free market operating in healthcare is undesirable can be traced back to Adam Smith,^99^ so successive marketisation reforms in the NHS have had to engage with exception mechanisms and taking different approaches to recognise the distinctive features of the healthcare sector.^100^ Certainly, the requirements in NHS policy guidance^101^ to distinguish effectively between ‘NHS patients’ on one hand, and ‘private patients’ on the other could seem to add an extra dimension to clinical decisions about treatment, which may become further complicated by the development of permutations within these (categories 2 and 3).

Examinations of ethical concerns with regard to NHS and private healthcare interaction have, perhaps unsurprisingly, focused on the expansion of private sector delivery of NHS services (thus category 2 activity) under New Labour^102^ and the HSCA 2012.^103^ This, logically, emphasises the organisational level, and introduces further nuance with distinctions between the ‘role expectations’ and the ‘ethical expectations’ of healthcare professionals, and the need to engage with the social realisation of ethics, whether we are interested in deciding what ought (or ought not) to be done by professionals or what ought (or ought not) to be done to professionals (by policy makers).^104^ Extrapolating this to the current discussion, the distinction between categories 2 and 3 becomes apparent and relevant, if not key. Category 2 activity (private sector delivery of NHS services) would require a focus on organisational ethics to factor in the solidarity underpinning NHS service delivery. In contrast, category 3 activity (NHS providers treating private patients) perhaps arguably requires greater focus on the clinical, rather than the organisational level, insofar as the solidarity aspect underpinning NHS care is removed, thus indicating a shift in mind-set.

While category 2 activity gives insights into ethical concerns and questions, the extension of these to wider interaction (and movement) between the NHS and private healthcare appears to attract less explicit attention. This is curious when it is recalled that the expansion of private sector delivery of NHS services has effectively been framed as a microcosm of wider NHS–private healthcare interaction.

Coexistence of the NHS and private healthcare sector, with the consequential option for patients to ‘go private’, appears to generate a range of ethical concerns, both from the perspective of patient behaviour,^105^ and that of healthcare providers^106^ in combining NHS and private work. Nevertheless, the concerns are pitched at the level of individual healthcare providers as to whether to accept exclusively NHS or private work, or whether to combine the two. However, while this might suggest a tendency towards the aforementioned ‘clinical level’,^107^ it is arguably ultimately concerned with the wider ‘organisational level’, which has been defined in law and policy since the inception of the NHS in 1948.

What also appears to link to the ‘organisational level’ is consideration of how treatments might be framed, and where distinctions may lie, since this may influence questions of rationing and access. Thus with dentistry, it may appear straightforward and uncontroversial to outline a distinction between treatments that are purely cosmetic, and those which treat, or at least seek to avoid, wider health problems. With IVF, this becomes more complex and controversial with considerations such as questioning the value of genetic parenthood^108^ on one hand, and the definition of infertility as a disease^109^ on the other. Such distinctions also raise positive questions about the relative scope for defining a private market for certain treatments, as well as normative questions about ethical concerns of doing so.

A final general consideration arising out of NHS–private healthcare interaction and its focus on the ‘organisational level’ is the effect on patients and how this may entail a shift, indeed a growing conflict, between individual and communitarian interests. This has been fuelled in part by the continuation of market-based reforms, culminating in the HSCA 2012. These reforms have also contributed directly to a sense of growing patient autonomy in connection with ‘patient choice’ policies,^110^ and indirectly to perhaps more subtle but fundamental challenges to the doctor–patient relationship in connection, for example, with information disclosure.^111^ Nevertheless, ‘patient choice’ policies naturally seem to emphasise the individual over communitarian interests given the linking in the NHS context of patient choice policies with marketisation reforms^112^ and the conceptualisation of patients as consumers able to opt for private treatment. This becomes more pronounced in the context of private healthcare and private medical insurance, with ethical concerns arising from the implication that scope for patients to rely on a general practitioner’s (GP) recommendation becomes restricted by a greater scope for self-referral.^113^ While this possibility may have evolved over time, it is receiving particular attention at the time of writing (January 2023), with Labour party proposals to expand self-referral by patients.^114^

### Specific considerations: ethical concerns and NHS–private healthcare interaction in response to COVID-19

Several of the considerations outlined above – particularly with regard to organisational ethics, for example – appear to have been replicated in the behaviour of private providers in connection with the agreements struck under the 2020 and 2022 Orders. Perhaps most obviously, the idea that some private providers would be more amenable to taking on NHS work than others was reaffirmed – with an evolving (and reducing) ‘core’ of providers joining the successive agreements. It is perhaps unsurprising that this should be reflected in both ‘crisis’ and ‘continuity’ responses.

Thus, the initial ‘crisis’ phase saw discussions both of a shift from an individual to a collective focus vis-à-vis access to COVID treatments (notably access to ventilators),^115^ and of whether the pandemic created a ‘paradigm shift’ in the ethical principles underpinning the NHS – away from justice-based principles to utilitarian values.^116^ The idea of a ‘continuity’ response can be linked not only to ensuring access to non-COVID services alongside the evolving pandemic, for example during the lockdowns, but also to the emerging – and ongoing – situation of addressing backlogs for various treatments which preceded, but were exacerbated by the pandemic. In this regard, important research has focused on the ethical challenges of ‘resetting’ specific services (such as maternity and paediatric care).^117^

The aforementioned ‘major deal’ struck in March 2020 is notable for setting a distinct tone for the initial phases of the pandemic. The deal was ‘unprecedented’ with the full range of private provision being redirected to supporting the NHS, albeit for a temporary period. The ‘crisis’ response during the initial lockdown – effectively a cessation of all private healthcare activity (in categories 3 and 4) – appeared significant for removing the long-standing ethical concerns about the coexistence of the NHS and private healthcare. However, the existence of the private healthcare continuity agreement^118^ quickly indicated a reversion to the status quo.

The use of PPEOs to give effect to the ‘major deal’, and to allow for subsequent relaxation of competition law by the 2022 Order, apparently in response to the emergence of the Omicron variant, indicates a certain degree of flexibility in ‘pausing’ the wider NHS–private healthcare competitive interaction. From an ethical perspective, this flexibility might suggest the persistence of the underlying ethical relationship – and focus on equity^119^ – as much as a shift in the ethical underpinning. The framing of a ‘healthcare disruption period’ with its legalistic and formalistic location within competition law, however, should raise concerns about the explicit linking of healthcare with market forces. This may offer an additional – or at least reinforced – dimension to the aforementioned research on organisational ethics, which is relevant beyond the scope of NHS competition reforms and in this wider NHS–private healthcare interaction.

## Concluding remarks

The interaction between public and private healthcare provision remains a difficult and contentious issue, and the particular experience of UK healthcare (albeit illustrated mainly by reference to England) provides some insights but raises more questions. The effects of responding to the COVID-19 pandemic cannot easily be grasped, but as at January 2023, we may start to draw distinctions between the ‘crisis’ phase of the initial outbreak in early 2020 and subsequent phases. These continue to see a refocusing of non-COVID services as the pandemic evolved to generate further surge periods. By examining the healthcare-related PPEOs – which might otherwise be dismissed as particularly niche aspects of competition law – it has been possible to provide an additional dimension to contemporary and perennial concerns, namely interaction between the NHS and private healthcare. This has generated at least three main insights.

First, while the flexibility of the PPEO mechanism may not herald significant change in and of itself when juxtaposed against framings such as path dependency, it nevertheless indicates scope for shifts in NHS and private healthcare interaction. This appears particularly evident in Wales, given suggestions of development of this interaction following closer cooperation between the NHS and private providers in the ‘crisis’ response.

Second, broad framings of ‘crisis’ and ‘continuity’ responses show the possibility of how examination of NHS–private healthcare interaction (and indeed other areas of law and policy, such as competition law) may complement considerations of ethical frameworks. In addition it highlights how future research may engage more with the diversity of aspects underpinning healthcare organisation, and how the ‘organisational level’ is as significant as the ‘clinical’ level.

Third, having an unusual level of public access to information about how private healthcare providers work with the NHS (with the agreements notified under the 2020 and 2022 Orders being published online) with regard to the COVID-19 pandemic helps indicate the diversity of NHS–private healthcare interaction. This also helps suggest where further research may be beneficial, both with regard to shaping future policy, and also in ethical considerations. It is important and useful to have greater awareness of the systemic levels of private healthcare involvement in NHS service delivery, as well as at the level of individual practitioners taking on a certain amount of private work alongside their NHS workloads.

Finally, that the COVID-19 responses may yet prove, at most, a contributory factor in – rather than the sole lever for – any subsequent significant changes to the deeply entrenched model of NHS–private healthcare interaction. As at January 2023, the NHS is beset by a range of problems, including lack of facilities and culminating in unprecedented strike action among healthcare professionals, so it may seem that the stage is set for some kind of significant change. The Sunak government’s strength may well be in question (relative to the support for the Thatcher government in the mid-1980s), but, importantly, the Labour party is showing itself not to be opposed to similar policy solutions involving private sector delivery of NHS services. These considerations may yet shape any development of the aforementioned Chope Bill.

## Footnotes

1. BBC News, ‘Dentistry: NHS Patients “Asked to Pay for Private Care’’’, 8 February 2021, available at https://www.bbc.co.uk/news/uk-55978595; BBC News, ‘Full Extent of NHS Dentistry Shortage Revealed by Far-Reaching BBC Research’, 8 August 2022, available at https://www.bbc.co.uk/news/health-62253893.

2. The Human Fertilisation and Embryology Authority (HFEA) indicated in 2020 that roughly 35% of IVF treatment in England is provided by the NHS, prompting further enquiry. See S. Smith and O. Marshall, *BPAS Investigation into the IVF Postcode Lottery: An Examination of CCG Policy for the Provision of Fertility Services*, August 2020, available at https://www.bpas.org/media/3369/bpas-fertility-ivf-postcode-lottery-report.pdf.

3. S. Germain, *Justice and Profit in Healthcare Law* (Oxford: Hart, 2019) pp. 10–11.

4. M. Guy, ‘Between “Going Private” and “NHS Privatisation”: Patient Choice, Competition Reforms and the Relationship between the NHS and Private Healthcare in England’, *Legal Studies* 39(3) (2019), p. 479.

5. S. Ost and H. Biggs, Exploitation, Ethics and Law: Violating the Ethos of the Doctor-Patient Relationship (Abingdon: Routledge, 2022), Chapter 3.

6. M. Guy, Competition Policy in Healthcare: Frontiers in Insurance-Based and Taxation-Funded Systems (Cambridge: Intersentia, 2019), Chapter 1.

7. NHS England, ‘News: NHS Strikes Major Deal to Expand Hospital Capacity to Battle Coronavirus’, 21 March 2020, available at https://www.england.nhs.uk/2020/03/nhs-strikes-major-deal-to-expand-hospital-capacity-to-battle-coronavirus/.

8. To give an indication of relative scope, the IHPN website lists 69 members in England, 14 members in Scotland and 12 members in Wales. See: https://www.ihpn.org.uk/membership/. Although no members are listed in Northern Ireland, it is clear that similar arrangements are in place – for example the Kingsbridge Private Hospital in Belfast provides information about its ‘NHS partnerships’. See: https://kingsbridgeprivatehospital.com/belfast/patient-information/nhs-partnerships/.

9. Similar arrangements appear to have been in place in Scotland and Northern Ireland, but not subject to the same formal notification procedures. For discussion of arrangements in these two countries, see British Medical Association (BMA), ‘Delivery of Healthcare during the Pandemic’, BMA Covid Review 3, 25 June 2022, p. 29. Such distinction can be attributed both to historically different approaches to the NHS across the UK as well as more recent devolution arrangements.

10. NHS England, ‘NHS Strikes New COVID Surge Deal with Independent Sector’, *News*, 10 January 2022, available at https://www.england.nhs.uk/2022/01/nhs-strikes-new-covid-surge-deal-with-independent-sector/.

11. Cabinet Office, COVID-19 Response: Autumn and Winter Plan 2021, available at https://www.gov.uk/government/publications/covid-19-response-autumn-and-winter-plan-2021/covid-19-response-autumn-and-winter-plan-2021#contents.

12. SI 2020 No. 368.

13. By the Competition Act 1998 (Health Services for Patients in Wales) (Coronavirus) (Public Policy Exclusion) Order 2020. SI 2020 No. 435. While NHS structures and relative extent of private healthcare provision differ across the UK, the Competition Act 1998 is UK-wide in its scope.

14. By the Competition Act 1998 (Coronavirus) (Public Policy Exclusions) (Revocations) Order 2021. SI 2021 No. 773.

15. NHS England, ‘Preparing the NHS for the Potential Effect of the Omicron Variant and Other Winter Pressures’, 13 December 2021, available at https://www.england.nhs.uk/coronavirus/publication/preparing-the-nhs-for-the-potential-impact-of-the-omicron-variant-and-other-winter-pressures/.

16. The Competition Act 1998 (Health Services for Patients in England) (Coronavirus) Public Policy Exclusion Order 2022. SI 2022 No. 124. This came into force on 9 March 2022 and expired on 31 March 2022.

17. C. Smyth, ‘Private Hospitals Could Ease NHS Backlog’, *The Times*, 8 December 2022. This is distinct from patients accessing private healthcare (as private patients), which generates separate concerns. See, for example, Engage Britain, ‘Millions Turning to Private Healthcare Forced into Financial Worry’, available at https://engagebritain.org/news-millions-turning-to-private-healthcare-forced-into-financial-worry/.

18. Indeed, this might be attributed to section 5 National Health Service Act 1946, which enabled consultants to continue private practice alongside their NHS workload. This was recognised in more general terms as the concession necessary to implement the NHS described in characteristically colourful terms by Aneurin Bevan as “stuffing their mouths with gold.” N. Timmins, *The Five Giants – A Biography of the Welfare State* (London: William Collins, 2017), p. 115.

19. Recent research to engage with this relationship, albeit within wider, comparative contexts, include Germain, *Justice and Profit in Healthcare Law*, and Guy, *Competition Policy in Healthcare*.

20. See, for example, in the Canadian context, C. Flood, ‘Two-Tier Healthcare after Cambie’, *Healthcare Management Forum*, 34(4) (2021), pp. 221–224, and in the Irish context, A. Murphy, J. Bourke and B. Turner, ‘A Two-Tiered Public-Private Health System: Who Stays in (Private) Hospitals in Ireland?’ *Health Policy* 124(7) (2020), pp. 765–771.

21. See, for example, BMA, ‘Delivery of Healthcare during the Pandemic’.

22. Guy, *Competition Policy in Healthcare*, p. 40, and developed from the relationships as set out in Office of Fair Trading (OFT), *Private Healthcare Market Study*, OFT1396, and O. Odudu, ‘Competition Law and the National Health Service’, *Competition Bulletin: Competition Law Views from Blackstone Chambers*, 8 October 2012, available at https://competitionbulletin.com/2012/10/08/competition-law-and-the-national-health-service/.

23. Ibid.

24. A further definition may include the “upgrade” of NHS Trusts to operate as Foundation Trusts, with greater autonomy from central government. See further, Guy, ‘Between “Going Private” and “NHS Privatisation”’ and Guy, *Competition Policy in Healthcare*.

25. See further, Department of Health, ‘Guidance on NHS Patients Who Wish to Pay for Additional Private Care’, 23 March 2009; Department of Health, ‘A Code of Conduct for Private Practice: Recommended Standards of Practice for NHS Consultants’, January 2004; NHS Commissioning Board (now NHS England), ‘Commissioning Policy: Defining the Boundaries between NHS and Private Healthcare’, NHSCB/CP/12, April 2013.

26. See further E. Jackson ‘Top-Up Payments for Expensive Cancer Drugs: Rationing, Fairness and the NHS’, *Modern Law Review* 73(3) (2010), p. 399; K. Syrett ‘Mixing Private and Public Treatment in the UK’s National Health Service: A Challenge to Core Constitutional Principles?’, *European Journal of Health Law* 17 (2010), p. 235.

27. W. Whittaker and S. Birch, ‘Provider Incentives and Access to Dental Care: Evaluating NHS Reforms in England’, *Social Science & Medicine* 75 (2012), pp. 2515–2521.

28. L. Laverty and R. Harris, ‘Can Conditional Health Policies Be Justified? A Policy Analysis of the New NHS Dental Contract Reforms’, *Social Science & Medicine* 207 (2018), pp. 46–54.

29. By Clinical Commissioning Groups (CCGs) established under the HSCA 2012. Following enactment of the HCA 2022, CCGs have been replaced by Integrated Care Boards.

30. HFEA, ‘Fertility Treatment 2019: Trends and Figures’, May 2021, available at https://www.hfea.gov.uk/aboutus/publications/research-and-data/fertility-treatment-2019-trends-and-figures/#Section8.

31. For example, Office of Fair Trading (OFT), Dentistry – An OFT Market Study, May 2012, OFT1414 and the recent consultation regarding IVF – CMA, ‘CMA Issues Draft Guidance on Consumer Law for IVF Sector’, *News Story*, 3 November 2020, available at https://www.gov.uk/government/news/cma-issues-draft-guidance-on-consumer-law-for-ivfsector.

32. CMA, Private Healthcare Market Investigation Final Report, CMA25, 2 April 2014, para. 2.15.

33. A number of private hospital groups recorded in their annual reports that the increased demand for private provision within the NHS and this new income from the NHS was used to compensate for falls in private patient numbers. See S. Arora, A. Charlesworth, E. Kelly and G. Stoye, ‘Public Payment and Private Provision – The Changing Landscape of Health Care in the 2000s’, Research Report (London: Institute for Fiscal Studies / Nuffield Trust, 2013), p. 30.

34. Section 2 Competition Act 1998.

35. See, respectively, OFT, ‘OFT Welcomes Action by NHS Trusts to Ensure Compliance with Competition Law’, OFT Press Release 71/12, 16 August 2012, and CMA, ’60-Second Summary: Private Medical Practitioners: Information on Competition Law’, 3 December 2015.

36. For further discussion, see Guy, *Competition Policy in Healthcare*.

37. One surgeon who works privately and in the NHS told The Independent: ‘Without the three-month contract during the crisis, private hospitals would have gone bankrupt as they had no business during the lockdown’. S. Lintern, ‘NHS on Life Support: Up to One in Six Will Be on Waiting Lists as Health Service Turns to Private Hospitals’, *The Independent*, 6 June 2020, available at https://www.independent.co.uk/news/health/coronavirus-nhs-waiting-timessurgery-privatisation-a9550831.html.

38. Competition Act 1998 Schedule 3 paragraphs 7(1) and (2).

39. The Competition Act 1998 (Groceries) (Coronavirus) (Public Policy Exclusion) Order 2020, 2020 No. 369.

40. The Competition Act 1998 (Dairy Products) (Coronavirus) (Public Policy Exclusion) Order 2020, 2020 No. 481.

41. The Competition Act 1998 (Solent Maritime Crossings) (Coronavirus) (Public Policy Exclusion) Order 2020, No. 370.

42. Emphasis added.

43. By the Competition Act 1998 (Coronavirus) (Public Policy Exclusions) (Revocations) Order 2021. 2021 No. 773.

44. B. Fakhruddin, K. Blanchard and D. Ragupathy, ‘Are We There Yet? The Transition from Response to Recovery for the COVID-19 Response’, *Progress in Disaster Science* 7 (2020), p. 100102.

45. D. Alfandre, V. A. Sharpe, C. Geppert, M. B. Foglia, K. Berkowitz, M. B. Chanko and T. Schonfeld, ‘Between Usual and Crisis Phases of a Public Health Emergency: The Mediating Role of Contingency Measures’, *The American Journal of Bioethics* 21(8) (2021), pp. 4–16.

46. P. Baines, H. Draper, A. Chiumento, S. Fovargue and L. Frith, ‘COVID-19 and Beyond: The Ethical Challenges of Resetting Health Services during and after Public Health Emergencies’, *Journal of Medical Ethics* 46(11) (2020), pp. 715–716.

47. L. Frith, H. Draper, S. Fovargue, P. Baines, C. Redhead and A. Chiumento, ‘Neither “Crisis Light” nor “Business As Usual”: Considering the Distinctive Ethical Issues Raised by the Contingency and Reset Phases of a Pandemic’, *The American Journal of Bioethics* 21(8) (2021), pp. 34–37.

48. Article 2, 2020 Order.

49. Article 5(2), 2020 Order.

50. Explanatory Memorandum to the Competition Act 1998 (Coronavirus) (Public Policy Exclusions) (Revocations) Order 2021, paragraphs 7.3 and 7.4.

51. Article 3(2) 2020 Order. See also Paragraph 7.4 Explanatory Memorandum to The Competition Act 1998 (Health Services for Patients in England) (Coronavirus) (Public Policy Exclusion) Order 2020, 2020 No. 368.

52. Explanatory Memorandum, paragraphs 7.2 and 7.3.

53. Section 3(1)(b) 2020 Order.

54. IHPN (HE01) Notification of 9 April 2020, available at https://assets.publishing.service.gov.uk/government/uploads/system/uploads/attachment_data/file/886123/he01-independent-healthcare-providers-network-notified-9-april-2020.pdf.

55. IHPN (HW01) Notification of 30 April 2020, available at https://assets.publishing.service.gov.uk/government/uploads/system/uploads/attachment_data/file/886125/hw01-independent-healthcare-providers-network-notified-30-april-2020.pdf.

56. 6 April 2020 in Wales and 15 April 2020 in England – IHPN (HE01) Notification of 9 April 2020 and IHPN (HW01) Notification of 30 April 2020.

57. IHPN (HE01) Notification of 9 April 2020 and IHPN (HW01) Notification of 30 April 2020.

58. See ‘IHPN England Members’, available at https://www.ihpn.org.uk/membership/england/.

59. It being noted that the agreement does not cover Mental Health Trusts, for example. See ‘Authorities and Trusts – NHS Trusts’, available at https://www.nhs.uk/servicedirectories/pages/nhstrustlisting.aspx.

60. See ‘IHPN Wales members’, available at https://www.ihpn.org.uk/membership/wiha/.

61. http://www.wales.nhs.uk/nhswalesaboutus/structure.

62. IHPN (HE02) Notification of 30 April 2020, available at https://assets.publishing.service.gov.uk/government/uploads/system/uploads/attachment_data/file/886124/he02-independent-healthcare-providers-network-notified-30-april-2020.pdf.

63. T. Norton, ‘NHS Triggers “De-Escalation” Clause to Allow Private Providers to Restart Work’, *Health Service Journal*, 15 May 2020, available at https://www.hsj.co.uk/coronavirus/nhs-triggers-de-escalation-clause-to-allow-privateproviders-to-restart-work/7027662.article.

64. Health and Social Care Committee, Delivering Core NHS and Care Services during the Pandemic and Beyond (HC 2019-21, HC320).

65. IHPN (HE03) Notification of 22 October 2020, available at https://assets.publishing.service.gov.uk/government/uploads/system/uploads/attachment_data/file/934591/independent-healthcare-providers-network-notification-22-october-2020.pdf.

66. IHPN (HE04) Notification of 16 December 2020, available at https://assets.publishing.service.gov.uk/government/uploads/system/uploads/attachment_data/file/953353/Independent_Healthcare_Providers_Network__HE04__-_notification_-_16_December_2020.pdf.

67. First identified in south-east England in September 2020. J. Chowdhury, S. Scarr, A. MacAskill and A. R. C. Marshall, ‘Variant of Concern – How a Deadly and More Contagious Variant of the Coronavirus Tore through the UK and across the World’, Reuters Graphics, 26 March 2021, available at https://graphics.reuters.com/HEALTHCORONAVIRUS/UK-VARIANT/ygdpzgblxvw/.

68. L. Dunhill, ‘Private Sector “Pushing Back” on NHS Request to Take More Patients, Say Top Trust’, *Health Service Journal*, 13 January 2021, and J. Rapson, ‘Independent Providers “Prioritising Private Patients over NHS”’, *Health Service Journal*, 10 August 2021.

69. In contrast, the private healthcare ‘continuity’ agreement enabled private patients to be treated during the wider ‘crisis’ response.

70. IHPN HE04.

71. IHPN HE05.

72. BMA, ‘Delivery of Healthcare during the Pandemic’.

73. Ibid.

74. Ibid.

75. Ibid, p. 28.

76. See, for example, I. Greener, ‘Understanding NHS Reform: The Policy-Transfer, Social Learning, and Path-Dependency Perspectives’, *Governance: An International Journal of Policy, Administration, and Institutions*, 15(2) (2002), pp. 161–183; and G. Bevan and R. Robinson, ‘The Interplay between Economic and Political Logics: Path Dependency in Health Care in England’, *Journal of Health Politics, Policy and Law* 30(1–2) (2005), pp. 53–78. More generally, see D. Wilsford, ‘Path Dependency, or Why History Makes It Difficult, But Not Impossible to Reform Health Care Systems in a Big Way’, *Journal of Public Policy* 14(3) (1994), pp. 251–283.

77. Greener, ‘Understanding NHS Reform’.

78. Wilsford, ‘Path Dependency, or Why History Makes It Difficult, But Not Impossible to Reform Health Care Systems in a Big Way’.

79. Section 5 National Health Service Act 1946.

80. Section 6 National Health Service Act 1946.

81. HC Deb 05 May 1975 vol 891 cc1084-149 HOSPITAL PAY BEDS (Hansard, 5 May 1975) (parliament.uk), available at https://api.parliament.uk/historic-hansard/commons/1975/may/05/hospital-pay-beds.

82. T. Blair ‘We Must Not Waste This Precious Period of Power’, speech given at South Camden Community College, London, 23 January 2003, cited in Z. Cooper, ‘Competition in Hospital Services’, OECD Working Party No 2 on Competition and Regulation (DAF/COMP/WP2(2012)2, 2012).

83. Greener, ‘Understanding NHS Reform’.

84. Ibid.

85. Arora et al., ‘Public Payment and Private Provision’.

86. Wilsford, ‘Path Dependency, or Why History Makes It Difficult, But Not Impossible to Reform Health Care Systems in a Big Way’.

87. Ibid.

88. Ibid.

89. Initially with the ‘NHS Five Year Forward View’, then the 2019 NHS Long Term Plan, which formed the basis for the HCA 2022.

90. See further in this context, M. Guy, ‘Dealing with ‘Unworkable Ideas in Primary Legislation’: Juridifying and Dejuridifying Competition in the English National Health Service’, *Public Law* January 2023, pp. 57–80, available at https://eprints.lancs.ac.uk/id/eprint/175079/.

91. Bill 105 2022-23. https://bills.parliament.uk/bills/3283.

92. The second reading is currently scheduled for 3 March 2023.

93. Initially used to explain shifts in US health care. J. W. Kingdon, *Agendas, Alternatives, and Public Policies*, 2nd ed. (Boston, MA: Pearson, 2014).

94. This has also been considered with regard to the EU-level responses to the pandemic. See M. Guy, ‘COVID-19: An Accelerating Force for EU Activity in Health?’, in S. Börner and M. Seeleib-Kaiser, eds., *European Social Policy and the COVID-19 Pandemic* (New York: Oxford University Press, 2023), pp. 246–271.

95. Lintern, ‘NHS on Life Support’.

96. R. T. Hall, *An Introduction to Healthcare Organizational Ethics* (New York: Oxford University Press, 2000), p. 4.

97. Ibid.

98. A. S. Relman, ‘Medical Professionalism in a Commercialized Healthcare Market’, *Journal of the American Medical Association* 298 (2007), pp. 2668–2670.

99. T. Zuiderent-Jerak, K. Grit and T. van der Grinten, ‘Markets and Public Values in Healthcare’, Institute of Health Policy and Management, Erasmus University Rotterdam, Working Paper W2010.01.

100. As evidenced by considerations of the extent of applicability of competition law and the development of the ‘relevant patient benefits’ concept in connection with merger assessment following the HSCA 2012 reforms. For further discussion, see Guy, *Competition Policy in Healthcare*, Chapter 2 regarding competition law, and Chapter 4 regarding mergers.

101. Department of Health, ‘Guidance on NHS Patients Who Wish to Pay for Additional Private Care’ and ‘A Code of Conduct for Private Practice’.

102. Notably the programmes of Independent Sector Treatment Centres. A. Cribb, ‘Organizational Reform and Health-Care Goods: Concerns about Marketization in the UK NHS’, *Journal of Medicine and Philosophy* 33 (2008), pp. 221–240.

103. L. Frith, ‘The NHS and Market Forces in Healthcare: The Need for Organisational Ethics’, *Journal of Medical Ethics* 39(1) (2013), pp. 17–21.

104. Cribb, ‘Organizational Reform and Health-Care Goods’, p. 229.

105. I implore people to stop using private healthcare: it’s killing the NHS | Jessica Arnold | *The Guardian*, available at https://www.theguardian.com/commentisfree/2020/jan/08/private-healthcare-sector-nhs-staff.

106. Private practice is unethical—and doctors should give it up | *The BMJ*, available at https://www.bmj.com/content/350/bmj.h2299; Private practice: is it unethical? (anthonygold.co.uk), available at https://anthonygold.co.uk/latest/blog/private-practice-is-it-unethical/; Private work has negative impact on NHS, expert says - BBC News, available at https://www.bbc.co.uk/news/health-32589521.

107. Although aspects of concerns at the ‘clinical level’ appear to remain constant – with questions of beneficence and non-maleficence within the doctor–patient relationship applying regardless of whether the doctor is treating an NHS patient or a private patient.

108. As but one value to be balanced against others, notably distributive justice (allocating public money to different goals) and non-maleficence (not harming patients and future offspring). See H. Mertes and G. Pennings, ‘Ethical Considerations of Fertility Preservation’, in M. Grynberg and P. Patrizio, eds., *Female and Male Fertility Preservation* (Cham: Springer, 2022), pp. 627–640.

109. Notably by the World Health Organization. WHO, Fact Sheets, Infertility, 14 September 2020, available at https://www.who.int/news-room/fact-sheets/detail/infertility.

110. See the varying interpretations of ‘patient choice’ policies, for example, I. Whiteman ‘The Fallacy of Choice in the Common Law and NHS Policy’, *Health Care Analysis* 21 (2013), p. 146 and M. K. Sheppard, ‘Fallacy or Functionality: Law and Policy of Patient Treatment Choice in the NHS’, *Health Care Analysis* 24 (2016), p. 279.

111. See comments by Lord Kerr about the more active role of patients who are increasingly treated as consumers in *Montgomery v Lanarkshire Health Board (General Medical Council intervening)* [2015] UKSC 11 at para 75.

112. Other framings are made between patient choice and patient empowerment, for instance in Nordic public healthcare systems. J. Tritter, M. Koivusalo, E. Ollila, P. Dorfman, Globalisation, Markets and Healthcare Policy – Redrawing the Patient as Consumer (Abingdon: Routledge 2011), Chapter 1.

113. Ethics at the interface—private practice and the NHS | *The BMJ*, available at https://www.bmj.com/content/330/7502/s215.2.

114. R. Sylvester, ‘Wes Streeting: We Must Think Radically – I Want to Phase Out the Existing GP System’, *The Times*, 6 January 2023.

115. C. Newdick, M. Sheehan and M. Dunn, ‘Tragic Choices in Intensive Care during the COVID-19 Pandemic: On Fairness, Consistency and Community’, *Journal of Medical Ethics* 46 (2020), pp. 646–651.

116. S. Germain, ‘Will COVID-19 Mark the End of an Egalitarian National Health Service?’ *European Journal of Risk Regulation* 11 (2020), pp. 358–365.

117. P. Baines, H. Draper, A. Chiumento, S. Fovargue and L. Frith, ‘COVID-19 and beyond: The Ethical Challenges of Resetting Health Services during and after Public Health Emergencies’, *Journal of Medical Ethics* 46(11) (2020), pp. 715–716.

118. IHPN (HE02), Notification of 30 April 2020.

119. Equity appears most relevant to the current discussion. Work done by the Royal Colleges indicates an underscoring of inequality in connection with resetting outpatient services. See The Royal College of Physicians and the Royal College of General Practitioners, ‘Rebuilding the NHS: Resetting Outpatient Services for the 21st Century in the Context of COVID-19’, Royal College of Physicians, 2020, available at https://www.rcplondon.ac.uk/guidelines-policy/rebuilding-nhs-resetting-outpatient-services-21st-century-context-covid-19. This can be contrasted with the focus of the Reset Ethics project where the focus on maternity services naturally underscores the significance of relational ethics.

